# Observations and Experiments on Pus

**Published:** 1811-04

**Authors:** George Pearson


### *Observations and Experiments on Pus.* By George Pearson, M. D. F. R. S.

(Phil. Trans. Part II. 1810.)

CHEMICAL writers vary in their statements of the properties of pus; and they consider that a further investigation is requisite for the purposes of science. Physicians confess that, in numerous cases, they cannot form a satisfactory judgment of the nature of diseases, on account of not being able to determine what is, and what is not purulent matter; likewise probably, on account of the existence of different kinds, or varieties, at least, of this substance, afforded by different disorders.

I beg leave, therefore, to submit to this learned Society, my own observations, experiments, and reasoning on this animal matter.

### Section I. *Simple and obvious Properties.*

The different kinds of fluid, commonly considered to be pus, may be distinguished by the following titles:

I. The cream-like and equally consistent.

II. The curdy and unequal in consistence.

III. The serous and thin kind.

IV. The thick, viscid or slimy.

1. A pint of the first sort was taken out of the pericardium, after a fatal inflammation of the heart, in St. George's Hospital, and obligingly sent to me by my colleague, Dr. E. N. Bancroft.

The *colour* was yellowish—the *smell* was fleshy when warmed—it was smooth and unctuous to the touch.

2. The specific gravity of two different portions, was as 1630 and 1633, that of distilled water being 1580; each substance stance being of the same temperature. Serum of the blood of different patients was found at the same time to be 1626, 1627, and 1630. Accordingly, the distilled water being 1000, the pus is 1031, and 1033, and the serum is 1029, and 1031.

3. After twelve hours repose, about two ounces by measure of a limpid fluid having appeared on the top, it was decanted from off the opaque purulent fluid; which was become thinner in the upper part of the vessel containing it, and thicker in the lower than before.

4. On further repose, it did not become offensive so soon as a portion of the same pus mixed with a little blood, nor as serum alone.

5. This pus neither indicated acidity nor alkalescency to the usual tests, viz. turnsole paper, tincture of red cabbage, Brazil-wood paper, and turmeric paper. I have, in other instances sometimes, observed acidity to be indicated by turnsole paper; but in none alkalescency, so long as the matter remained without fœtor.

6. Being examined under the microscope, when duly diluted with distilled water, innumerable spherical particles were seen, which did not appear altered in figure, nor diminished in number by extreme dilution; that is, they did not appear to have been dissolved.

II. A pint of pus of the second kind, *viz. curdy*, was afforded by a psoas abscess.

The colour was brown. It felt knotty. On pouring from one vessel to another, the curdy masses were manifest, and of various sizes, from that of a pin's head to a hazel nut. It was more viscid than the former, and of a little greater specific gravity. On standing, a limpid fluid appeared upon the top, as in the first kind, but in smaller quantity. Globules were seen with the microscope, but also a number of irregularly figured larger masses. Putrefaction took place sooner than in the former kind. In other properties, this pus was similar to the first kind.

III. *Serous thin pus.* It was produced by a fatal inflammation of the peritoneal coat, without ulcer, and taken out of the cavity of the abdomen. A good deal of serum was also effused, of which the pus was a deposit. It was not much thicker than milk. To the feeling it was not at all unctuous. The smell was slightly offensive. On standing twenty-four hours a sediment appeared, occupying only one half the full vessel, under a whey-like liquid. Putrefaction took place sooner than in either of the two former kinds. The specific gravity was the same as that of the first sort.

In

In other properties it was similar to the cream-like pus above distinguished.

IV. A pint of the *viscid pus* was obtained from an abscess among the muscles of the thigh. If I had not had entire confidence in Mr. Brodie's accuracy, who was so obliging as to attend to my request, on this and many other like occasions, I should have supposed, that this was expectorated matter, it so exactly resembled in its simple properties, the *ropy kind*, described in a paper on expectorated matter. Phil Trans. 1809, P. II. p. 317.

The appearance was not quite uniform, there being semi-transparent masses in small proportion, mixed with the perfectly opaque white matter. It was almost inodorous. To the touch it was quite smooth. The specific gravity was nearly that of the second kind of pus.

On standing 24 hours, about one ounce measure of limpid fluid rose to the top of the whole mass. Putrefaction did not take place so soon as in expectorated matter of the same consistence.

The examination by the microscope manifested innumerable spherical particles among leafy masses, and numerous particles of irregular forms.

The simple properties were otherwise similar to those of the other sorts of pus, above distinguished.

Many other differences of purulent matter are universally recognized ; but they are either varieties of the four kinds already named, or the differences depend upon the obvious mixture with adventitious substances ; such as the red part of the blood, coagulated lymph, serum, putrefied matter, fibrous and membranous masses, calculi, &c. : therefore, I deem it useless to describe them.

### Sect. II. *Agency of Caloric.*

1. The above kinds of pus coagulated like serum of blood, into a firm, uniform, soft solid, at the temperature of 165°. completely ; but partially at 160° of Fahrenheit's thermometer.

2. The decanted limpid fluid from pus, Sect. I.—I. II. III. IV. coagulated completely into a firm uniform mass, like serum of blood, at 165°, but it became opaque and thickened at 160°. By pressure on the firm curd thus produced, a watery liquid was separated, which on due evaporation did not give a jelly, but was coagulable like the decanted liquid just mentioned.

The thick opaque matter, after decanting the limpid fluid, coagulated as before said, into a firm mass at 165°.

3. Each

3. Each of the above four kinds of pus being evaporated to dryness, left in no case less than one tenth of its original weight, nor more than one sixth; but most frequently one seventh, to one eighth of brittle matter. The smallest proportion of residue was left by the third, or serous kind; the largest, by the 2d or curdy. These residues generally became rather soft, especially those of the third, or the serous kind, after exposure to the air.

4. The opaque part of pus after separating the limpid fluid afforded on evaporation about $\frac{1}{15}$ to $\frac{1}{30}$ more of brittle residue, than an equal weight of the pus itself; and it remained hard on exposure to the air. The limpid fluid evaporated to dryness, yielded about one tenth of brittle residue; which grew moist, and sometimes deliquesced, on exposure to the air.

5. The brittle residues above mentioned (3), being exposed to fire in platina crucibles, flamed for some time, emitting a very offensive, pungent, empyreumatic smell; the uninflammable residue being kept in a state of ignition for a longer period, what remained at length was fused readily from the *serous*, *viz.* the third kind of pus; but in the cases of the other exsiccated residues of the 1st, 2d, and 4th kinds of pus, they barely were melted, or only became soft and claggy. The fused residues from the *serous pus* amounted to $\frac{1}{30}$ to $\frac{1}{35}$ of the exsiccated pus; and to $\frac{1}{250}$ to $\frac{1}{300}$ of the original purulent matter. Those from the second kind, the *curdy*, amounted to $\frac{1}{50}$ to $\frac{1}{60}$ of the dried matter, and to $\frac{1}{350}$ to $\frac{1}{400}$ of the pus itself. The fused masses from the 1st and 4th kinds of purulent matter, afforded intermediate quantities of melted matter between those just mentioned.

6. The fused residues (5), being treated in the manner described in a former paper, Phil. Trans. 1809. P. II. p. 326—329, I found they consisted chiefly of muriate of soda, phosphate of lime, and potash; with strong indications of carbonate of lime, and a sulphate; besides traces of phosphate of magnesia, oxide of iron, and vitrifiable matter, probably silica. On a reasonable calculation, it appeared, that in the *serous kind* of pus, the muriate of soda amounts to from one and a half, to two per 1000; the phosphate of lime to one, to one and a half per 1000; the potash to one half, to three fourths of a part in this quantity; and the other matters together, to half a part in 1000. In the *curdy matter*, the second kind, the muriate of soda amounts to three fourths of a part, to one in a 1000; the phosphate of lime to one; the potash to less than one half; and the other matters united, to half a part in a 1000. The first kind of pus, the *cream-like*, and the fourth, the *viscid*, afforded from the melted residue, the same substances as the *serous* kind, excepting a somewhat smaller portion of muriate of soda, and potash.

7. The brittle residues of evaporated pus, after decanting the limpid fluid (4), being treated with fire as above related, the remaining matters were melted with more difficulty, and less completely, and contained a smaller proportion of muriate of soda and potash than the original pus.

8. The decanted limpid fluids (4), being evaporated to dryness, these residues were exposed to fire. They were melted, and then afforded a larger proportion of muriate of soda and of potash, than the pus itself; but with the same proportion of the other saline and earthy substances.

### Sect. III. *Agency of Water.*

1. After decanting the limpid fluid from off half a pint of the four kinds of pus as above related, (Sect. I.) three ounces measure of distilled water were mixed with each of them. After 48 hours repose, a limpid fluid of nearly the quantity of two ounces by measure, was seen forming an upper stratum to the pus. It was decanted for examination.

(*a*) On exposure to fire it became turbid like milk, as soon as the temperature was elevated to 165°, but did not become thicker at a greater elevation.

(*b*) On evaporation to dryness, the residue amounted to about one fifteenth of the weight of the liquid from the serous pus, and to one twentieth from the three other kinds; in place of about one tenth, as from the first decanted liquid, (Sect. I. 4); and as from serum of blood. The residuary matters were of the same kind as those above described, Sect. II. 2—6.

(*c*) Three ounces measure of distilled water having been again mixed with each of the four kinds of pus, and in 48 hours, two ounces measure of decanted limpid fluid from each, having been evaporated to dryness, residues of the same kind, in the same proportions, and in nearly the same quantities as before, were obtained (*b*). These decanted fluids became nearly as turbid as the former, on raising their temperature to 165°.

(*d*) Distilled water was added a third time in the quantity of eight ounces by measure, to each of the four parcels of pus under examination, and after 48 hours repose, six ounces of limpid fluid were poured off from each of them. At the temperature of 165°, the decanted fluids became turbid; that of the serous pus more so than the others. On evaporation to dryness, a much smaller quantity of residue was obtained than than before, *viz.* one sixtieth from the serous pus, and one seventieth from the others; and it consisted of the same kind of substances as above described; but the muriate of soda and potash were in smaller proportion than before.

(*e*) A fourth time distilled water in the quantity of a pint, was mixed with the present four parcels of pus, and after standing 48 hours, three fourths of a pint of clear colourless liquid was poured off from each of them. It became slightly turbid and whitish on boiling. On evaporation, each parcel afforded about $\frac{1}{90}$ of the fluid employed. The residues now consisted of animal matter, with a much smaller proportion than before, of muriate of soda, phosphate of lime, and potash—nothing else could now be traced.

(*f*) Distilled water in the quantity of a pint, was once more mixed with the four sorts of purulent matter undergoing inquiry. After 48 hours, a pint of liquid was descanted from off each of them; but being slightly turbid, they were left to stand 24 hours. By this time a sediment was deposited from each of the liquors; but being still, though very slightly, turbid, they were filtrated through suitable paper. They were then transparent. The transparent filtrated liquors had their transparency disturbed by a boiling temperature. They became also slightly milky, with nitrate of silver, but scarcely so with infusion of gall nut. On evaporation to the quantity of an ounce from each pint, the residuary liquids appeared slightly globular. These on evaporation to dryness, yielded not more than one part of animal matter, from each 500 of the transparent filtrated liquids.

(*g*) On standing three or four days in a cold room, the parcels of pus, after the ablutions just related (*a*—*f*), exhibited a whey coloured liquor at the top, of which about $\frac{1}{4}$ of a pint was poured off from them. More turbid liquor was also separated from the washed pus, by pouring it upon a porous cotton cloth strainer, which left purulent matter of the consistence of starch mucilage, amounting to but one half the original weight.

(*h*) The pus freed from coagulable limpid liquid by repeated ablutions (*a*—*h*) was white as snow—equal in consistence—perfectly smooth—the 4th kind was less viscid than before, but the others were more so—no smell—not at all disposed to putrefy—on elevating its temperature to 165° and higher, it did not coagulate into one mass, nor into clots, or large masses of curd, but a watery fluid separated from a fine soft somewhat curd-like opaque fluid; which did not become more curdy, on even boiling—it did not appear that above a grain of this part, or state of pus, dissolved in 1000 waters—was highly globular under the microscope, and remained so,

 although although coagulated by nitrate of silver; by infusion of gall nut; by alcohol; and super-sulphate of alumina—with muritate of ammonia, nitrate of potash, and other neutral salts, and with carbonate of potash, it produced a viscid semi-transparent mass like expectorated half transparent matter—exposed to fire in a platina crucible, it was inflamed, but did not emit an offensive smell, and after continuing the ignition, the residue was a particle of half fused matter, not amounting to $\frac{1}{1000}$ of the pus after ablution, nor above $\frac{1}{500}$ of the same matter exsiccated; it consisted of phosphate of lime and vitrified matter—no ammonia was perceivable, on mixing lime with this washed pus; nor muriatic acid on adding sulphuric acid.

2. (*a*) A tea spoonful of the *cream-like pus*, being agitated in half a pint of distilled water, produced a milky fluid, with a number of small curdy particles suspended, but very few leafy or fibrous pieces or clots.

(*b*) The *serous pus* being treated as just mentioned (*a*), the same appearances ensued.

(*c*) The *curdy pus* being agitated in the same manner in water, a number of clots, leafy, and fibrous masses, were seen suspended among fine small curdy particles in a pearly liquid.

(*d*) The *viscid pus* being treated as just said, it required long continued, and violent agitation, to diffuse it through the water, and then the appearances were as last described.

3. Pus of any kind, after boiling in twenty times its quantity of water, was quite as globular under the microscope as previously. With a smaller proportion of water, the mixture became very turbid, sometimes clots were formed in a pearl liquid, in which a fine sediment took place, which appeared much more globular than the clots or curdy masses.

4. In general, water in which pus has been agitated, remains somewhat milky, with an abundant close white sediment; but after two, or three, or more ablutions, the water becomes clear on standing, and the sediment more curdy.

### Sect. IV. *Agency of Alcohol of Wine.*

The different kinds of exsiccated pus exposed to the agency of this menstruum, and treated as described in a former paper, Phil. Trans. 1809, P. II. p. 329, the results were similar, except in the proportion of products.

1. These exsiccated substances afforded to this menstruum a smaller proportion of potash, but as much animal oxide and muriate of soda, as mucous sputum.

2. The undissolved matter left after repeated digestions in this this menstruum, afforded the same substances, but in smaller proportions, as mucous sputum.

3. Equal bulks of fresh pus, and rectified spirit of wine, afford a much thicker and more milky liquor, with a closer sediment, than expectorated mucous matter.

SECT. V. *Agency of acetous Acid.*

The purulent matters mixed with this acid became curdy, and rendered it milky; but on standing, a close white sediment appeared, the liquid above being clear, except in the case of the viscid pus, which exhibited leafy and fibrous masses, as hath been described with mucous sputum.

By repeated digestion of the different kinds of pus in this menstruum, I obtained the same results, except the proportions of acetite of potash, and muriate of soda being smaller, as related in a former paper on mucous expectorated matter. Phil. Trans. 1809, P. II. p. 336.

SECT. VI.—*Some Experiments with different Objects, especially to distinguish Pus and Mucus.*

1. In the agency of sulphuric, nitric, and muriatic acids, in sufficient quantity to dissolve and decompound the substances under inquiry, I could perceive no important difference between them. The purulent matters indeed required a much greater proportion to completely dissolve them, than the transparent sputum. Also the more opaque and dense the sputum, the greater the resistance to dissolution. Sulphuric acid produced black liquids like those containing charcoal, smelling strongly of muriatic acid, but on dilution with water, they became clear. No precipitation occured on dilution with water, and on saturation with the fixed alkalies, but a trifling sediment appeared, which re-dissolved on the addition of the above acids.

2. The mineral acids diluted, or added in small proportion, and the vegetable acids, coagulate variously pus and mucous fluids. Some become merely milky fluids, others curdy fluids, others afford fibrous and leafy masses in a transparent liquor, and others give an uniform thick mass of curd. On standing the deposits are accordingly of various forms, and the liquors above of various appearances, but I could discover no constant characteristic property of the substances by these experiments, as some writers have asserted.

3. The solid fixed alkalies, or lime, mixed with expectorated mucus, occasion a stronger smell of ammonia than with pus; or with muco-purulent sputum. Some use may be perhaps perhaps made of this easy experiment to judge of the nature of varieties of the fluids in question, particularly as far as depends on the proportion of ammonia: for sometimes it cannot be perceived by the smell on mixing alkalies, but can by muriatic acid giving white vapours. Concentrated liquid alkalies, added to both pus and mucus, dissolve them to produce clear liquids, except small curdy parts and motes. These curdy parts and motes resist dissolution also for some time even in nitric acid, and seem to be self-coagulated lymph. They are in much greater proportion in pus than mucus. The addition of acids to these alkaline dissolutions occasions precipitations, but no differences, or not with sufficient uniformity to afford criteria, were observed according to the observations of other Experimenters.

4. Concentrated aqueous solutions of various neutral salts, *viz.* muriate of ammonia; nitrate of potash; muriate of soda; sulphate of soda, &c. being mixed in due quantity with pus of the kinds under examination, produce viscidity, like ropy expectorated matter, thickening like jelly, and less opacity. These changes have, in the case of muriate of ammonia, been called coagulation by Mr. HUNTER; but by agitation in cold water the matters are diffused, and on standing, the pus is precipitated in its original state. I call these effects of the neutral salts inspissation, seemingly occasioned by their attracting water from the pus; for no such change is produced if either the purulent matter, or solution of salts be diluted; nor is it produced if the pus be previously coagulated by caloric; also the inspissated pus is coagulated by caloric as usual. No such inspissation is produced by these salts in mucous sputum, nor in muco-purulent sputum, so that undoubtedly it is a criterion as discovered by Mr. HUNTER in the case of muriate of ammonia, and with other neutral salts, as now manifested.

4. I endeavoured to find some easy tests for distinguishing pus from mucus; but I did not succeed with the tanning principle; gallic acid; super sulphate of alumina; nitrate of silver, and other metallic salts; and as already said, various acids. They all produced precipitation of these animal matters, but not with observable characteristic differences.

5. To observe the state in which the matter of pus is secreted, I procured the assistance of Mr. MAYNARD, the present house-surgeon of St. George's hospital, and Mr. GEORGE EWBANK, who had been on several occasions essentially serviceable in my enquiries. Square pieces of gold beater's skin were applied to various sore legs after carefully removing the matter already secreted. In five to ten minutes the square pieces being removed, were found wet with a limpid limpid fluid. In this state they were inspected by the microscope, by which numerous globules were seen. In ten minutes further the liquid was no longer limpid but opaque like pus, in which the usual spherical particles were seen with the microscope as just mentioned.

Supposing objections might be offered on account of the alteration of texture of the skin employed, square pieces of glass were also applied. The results were the same in both trials. The two gentlemen above named, as well as Dr. Richard Harrison, and other pupils, who happened to be present, all concurred in the observation that the limpid matter became opaque, and that while limpid it was, like pus, full of spherical particles.

### Sect. VII. *Conclusions.*

The statement of the properties of pus in the foregoing inquiry I hope will be found to be true; and I submit to the judgment of others whether or no the following inferences are legitimately established.

1. That this fluid essentially consists of three distinct substances, viz. 1. An animal oxide, which, among other properties, is distinguished by its being white, opaque, smooth, of the form of fine curdy particles in water; not dissoluble in less than 1000 cold waters; not coagulable into one mass like serum of blood by caloric, alcohol, &c.; only rendered more curdy by water of 160° to 170°; but readily diffusible.—2. A limpid fluid resembling serum of the blood in its impregnations and in its coagulability by caloric, alcohol &c.; in which the opaque oxide is diffusible but not dissoluble, and which is specifically lighter than that oxide.—3. Innumerable spherical particles visible only by the microscope in this opaque oxide, and in small number in the limpid fluid; not coagulable by any temperature to which hitherto exposed, and not destructible by many things which combine or destroy the opaque oxide; and these globules are specifically heavier than water*.

2. That the *visible* curdy masses, as well as the fibrous or leafy parts, almost always contained in smaller or larger quantities in pus, may be considered as self-coagulated lymph, which in its fluid state is secreted without having the state of aggregation produced in it like that of the *essential* opaque oxide of pus.—Sect VII. 1.

* My obligingly attentive pupils, Mr. Burton, and Mr. Stansfeld, house-surgeons of the Lock hospital, collected for me a sufficient quantity of gonorrheal matter to determine that it consisted of the three ingredients here stated.

3. That

3. That the reddish, the blackish, and the dark brown colour of pus depends upon the red part of the blood effused or secreted from the same vessels, or from contiguous ones which secrete pus.

4. That on some occasions the clotty and irregular figured masses found in the pus may depend upon disorganization or breach of the contiguous solid parts.

5. That whenever pus is fœtid to the smell, a portion of it is in a state of putrefactive fermentation, which may be removed by ablutions with water.

6. That there are certain adventitious matters liable to be contained in pus not hitherto rendered palpable to the senses, but known by their effects in exciting contagious diseases; such as small-pox, syphilis, &c. These matters are produced by a specific action in the secretory organs of pus, by such matters themselves either contained in the circulating blood, or on the secreting surface.

7. That the *essential* substances of which pus consists, as well as some of the adventitious ones (Sect. VII. 1, 2, 3, 6), are separated from the blood by a peculiar organization belonging, or attached to the blood-vessels: which organs of separation or secretion are not only excited to the action which produces pus in diseased states, but they are evidently influenced by the states of other distant organs of the animal œconomy; hence many varieties in the properties of the purulent matter.

8. That the varieties of purulent matter relate to differences of *quantity*—the proportion of the essential substances (1)—and the adventitious parts (2, 3, 4, 5, 6,). The *cream-like* consisting of almost purely the opaque oxide and limpid liquid (I. 1, 2). The *curdy* containing a large proportion of coagulated lymph, or broken down solids. The *serous* abounding in limpid fluid. The *viscid* depending upon the coagulation, and perhaps, inspissation, by union of neutral salts with the opaque oxide.

9. That as the essential parts are secreted in a limpid state, but presently become opaque, owing to a large proportion spontaneously coagulating, and thus becoming the opaque oxide, mixed with the serous liquid, and innumerable spherical particles (Sect. VII. I. 1, 2, 3), it seems reasonable to infer that these matters are the self-coagulated lymph of the blood and serum, separated by the secretory organs; which act of secretion determines the subsequent state of aggregation of pus, and the globules are at the same time formed analogously to their formation by other secretory organs. How far they are those of the blood altered by secretion may be determined hereafter. It is a collateral proof of this inference that that very thick pus affords one sixth to one seventh of exsiccated brittle residue, which, as I have found, is nearly the same proportion afforded on the exsiccation of the buffy coat of inflamed blood; while very thin pus affords on exsiccation one eighth to one eleventh of brittle residue, which is the proportion to be expected from a mixture of serum of blood and self-coagulated lymph, as I have ascertained.

10. That the constant impregnating saline and earthy ingredients of pus are dissolved in the serous fluid, and are all separable along with the serum, by ablutions with water, from the opaque oxide (1), except a portion of the phosphate of lime. These impregnations are the same as those of serum of blood, and of expectorated mucous matter, *viz.* muriate of soda; potash neutralized by animal matter or a destructible acid; phosphate of lime; ammonia neutralized probably by phosphoric acid; with a sulphate and traces of some other matters mentioned in my former paper. The proportion of these impregnating substances is as the proportion of limpid or serous coagulable fluid, and of course inversely as the proportion of the opaque oxide of pus; but it varies in different cases in given proportions of this oxide, and the limpid fluid. In general, if not always, a given quantity of pus contains a smaller proportion of saline matters than an equal given quantity of expectorated mucous matter, but a given quantity of the limpid coagulable fluid contains a greater proportion of saline matters than an equal given quantity of serum of blood. Hence the thicker the pus the less irritation to the sore which secretes it, and commonly the less the inflammatory or other action of the secreting surface. In different cases, however, the proportion of impregnating saline substances to one another is liable to vary, especially that of phosphate of lime: hence, though rarely, calculi occur of this substance in the cavity of the abscess*. Hence too the exsiccated pus is liable to become soft and moist, from the proportion of neutralized potash being greater than usual; and even deliquescence sometimes occurs of the exsiccated limpid fluid.

12. That the same organs, according to their different states,

* On examining the lungs of a patient who died of pulmonary consumption, concretions were found in a large vomica from the size of a mustard seed to a pepper corn, which Dr. E. N. Bancroft reserved for my enquiry. I found they consisted chiefly of phosphate of lime with an unusually small proportion of animal matter. In another patient of Dr. Nevinson, matter was coughed up, consisting chiefly of phosphate of lime and animal matter, nearly one of the former to three of the latter.

secrete from the blood merely water impregnated with the saline substances of the serum of blood ; also this fluid containing various proportions of coagulable matter like that of serum of blood ; and serous fluid with self-coagulable lymph, which affords curdy masses : likewise this serous fluid, together with this matter which coagulates of itself after secretion, highly impregnated with invisible small particles, in such a state of aggregation, as to constitute the thick opaque fluid called pus—which states of the secretory organs are generally attended with inflammatory action, but frequently also without any symptoms of such action.

13. That besides the consistence of pus depending upon the proportion of serous limpid liquid, and opaque matter, it also probably depends upon the mode and state of coagulation of the matter which affords this opaque part; analogously to the different states of consistence of the coagulated blood itself, according to the different conditions of the animal œconomy.

According to the above inferences, I trust, a distinct and definite notion of the substance to be considered as pus is exhibited; and I do not comment on the different results of experiments and conclusions of other writers, because future observers only can determine the truth. What is and what is not pus will now readily be ascertained by a few easy experiments; by the obvious properties; and by the consideration of the source of the matter in question : provided, however, that it be unmixed with certain other matters by which disguise is produced. As already observed, it is in pulmonic diseases that the ambiguity occurs ; and physicians lay very considerable stress upon the nature of expectorated matter in their practice and reasoning ; I shall therefore endeavour to elucidate the subject by remarks on the puriform matter expectorated in different cases.

1. An abscess occasioned by acute inflammation not only of a pleurisy, and peripneumony, but of other diseases which have not the symptoms of any one which has received a designation. Here there ought to be no doubt ; for the matter which is coughed up suddenly and abundantly on the bursting of the abscess is evidently pus with little mucus. Such matter consists of the essential ingredients of pus, (Sect. VII. 1,) with generally adventitious substances, (Sect. VII. 2, 3, 4,) viz. coagulated lymph, membranous or fibrous parts, and a small portion of the red part of blood.

2. Purulent expectoration from the rupture of abscesses, or vomicæ of suppurated tubercles. In such cases there has been a chronical cough with viscid sputum, commonly in persons of an advanced age. After this long continued disease, ease, and abundant expectoration of quite a different kind from the former suddenly comes on; by which the patient often dies very speedily; sometimes immediately, being seemingly choaked. This kind of matter evidently consists chiefly of the essential ingredients of pus (Sect. VII. 1,) with not only the adventitious substances, viz. clots of self-coagulated lymph, and sometimes the red part of blood, but also masses, which are apparently the broken down solid parts, the cellular membrane, the vessels, and substance of the tubercles, in a disorganized state. The sufferer often says, such matter tastes sweet. The mucus is here in too small a proportion, and not intimately mixed, to occasion disguise.

3. In the bronchitis, or inflammatory affection of the air-tubes, the membrane remaining entire, attending various diseases, e. g. the measles, a fever with a cold, various continued fevers, an expectoration of thin cream-like matter occurs, at first gradually, but at last in great quantities, continuing for a week or more. Although mucus is usually coughed up with this puriform substance, the two things generally remain in distinctly large masses. With little skill, the opaque or puriform fluid may be collected separately from the mucous matter. It will be found to consist almost purely of the three essential constituents of pus (Sect. VII. 1,) there being seldom any adventitious substances.

4. Muco-purulent, or commixed expectorated matter. This kind is perhaps of the most frequent occurrence. It is that which many physicians know not how to designate; some consider it to be pus, and others to be mucous matter. This contrariety of opinion arises from the want of definite notions of pus and mucus. Hence the parties are not able to perceive that in this kind of sputum, exist many of the properties of pus, and also of mucus. I have described it in my former paper on expectorated matter, Phil. Trans. 1809, P. II. p. 317, under the denomination of *opaque ropy matter*, the third kind. I feel no degradation in finding it necessary to confess, that a better acquaintance with the properties of pus has taught me that I was in an error, in considering this kind of expectorated matter to differ from other sorts, merely in the proportion, and not in the kinds of constituent parts. It now appears, that the sputum in question possesses such properties as might be predicted to exist, from the known properties of pus and mucus separately, in case these two substances should be intimately commixed. Accordingly, the opacity; the straw colour; the greater density than mucus; the great globularity under the microscope;

 the the greater proportion of residue on evaporation to dryness, than from mucus ; the milky liquid on heating this matter ; the milkiness on agitation in cold water ; are properties of pus. But the great viscidity, yet not increased by neutral salts ; the less opacity than pus ; the less globularity than pus ; the smaller proportion of exsiccated residue than from pus ; the moisture, or greater moisture on the exposure of the brittle residue to air, than from that of pus ; the more difficult diffusibility through cold water, and less degree of milkiness than from pus ; the great proportion of leafy or fibrous masses on agitation in a very large quantity of cold water ; the speedy putrescency ; are properties of mucus. The mode of coagulation by caloric at 160° and upwards, is such as might be expected from the commixture, viz. in large masses of curd in a milky liquid, instead of into one uniform mass like pus, or into small curdy masses in a very large proportion of whey-coloured liquid, like mucous sputum. Thick pus affords on evaporation to brittleness, $\frac{1}{7}$ or $\frac{1}{8}$ residue ; and transparent sputum of the consistence of jelly, gives about $\frac{1}{13}$ or $\frac{1}{20}$ of such residue ; but this opaque matter under inquiry, affords $\frac{1}{10}$ to $\frac{1}{15}$ of brittle residue, according to the proportion of the two substances. I could not separate the supposed pus and mucus from one another, to exhibit them distinctly by water, or by any other means, on account, as I conceive, of the intimate diffusion through one another, and their mutual cohesion. But on evaporating the milky water, produced by agitating the sputum in it, or by letting it stand to collect the sediment, little else beside a mere congeries of globules seen under the microscope, was thus obtained. For the same reason, on standing, a serous liquid like that of pus (Sect. VII. 1,) does not separate, or only partially, from the opaque part, so as to render it possible by ablution, to collect this coagulable liquid like that of pus : and the greater proportion of water, belonging to the mucus, occasions the coagulation by caloric, to afford only a milky liquid, instead of an uniform mass of curd.

This kind of sputum, consistently with the phenomena, must be produced by secretion from the bronchial membrane in its entire state, and not by ulceration or abscess. For it is secreted in many cases, at the rate of a pint or more in each 24 hours, for weeks and months successively, and for 20 or more successive winters. Also many persons recover their good health after this secretion, and it is the usual termination favourably of pneumonia, bronchitis, &c. It is produced by any disease of great irritation of the lungs ; as I have found from ossification of the bronchial or pulmonary arteries ; ries; from calculi; from broken wind, or rupture of air cells, &c.*

It is secreted also in consequence of irritation of the bronchial membrane by tubercles, vomicæ, water in the cavities of the chest, &c. The same kind of matter is secreted from the nose on the decline of a common severe coryza in many cases. It appears then, that this kind of matter is a symptom of the most fatal, as well as harmless diseases—it is a symptom in one case, of the progress of disease to death, and in another, of the termination in health, by being seemingly a critical discharge. Perhaps, if these facts had been observed and considered, numerous mistakes in prognostics would have been avoided, and better practice have been employed; because the nature of diseases would have been rightly understood. From this representation, it is plain, that a just opinion cannot be given merely from the examination of the sputum, without considering the disease by which it is produced, or of which it is a symptom.

The proportion must also be considered of the pus and mucus in sputum; it may be estimated, by attending to the properties of each above stated.

Such a compound as the present, scarcely is produced in any other part, but in the bronchial, and mucous membrane of the nose, because of the abundant secretion of mucus from these membranes. And when it is conceived, that both pus and mucus are secreted in a limpid state, from the same or at least contiguous organs, where they first intimately commix, and then become inspissated; it will appear reasonable, that they cannot be readily, or at all completely separated again from one another. There is indeed, in these cases, no necessity for the admission of the secretion of the limpid fluid of pus of abscesses (Sect. VII. 1); for it appears to me not unjust to consider mucus to be nothing more than the serum of blood, altered in its composition and proportion of water, so as to produce a viscid texture. The secretory organs of the mucous membrane, by virtue of their peculiar power, separate from the blood, in health, the mucus as above said, with some globules, and also a small proportion of the self-coagulable lymph; which appears, on agitating mucus in a large proportion of cold water, in the form of leafy and fibrous masses†. The same secretory organs, it is easily conceivable,

---

* I believe this state of the lungs to have been first discovered in broken-winded horses, by Mr. Colman.

† Serum of blood appears always to contain self-coagulated lymph, which is deposited on standing; and this appearance led Gaber, Pringle, and Cullen, into the erroneous opinion, of this deposit being pus itself.

may in a diseased state be excited to separate also self-coagulable matter from the blood, with more globules, in such a state as to become pus. Hence, such a commixture of the two substances must correspond to the opaque, viscid, expectorated sputum of which I am writing.

If I thought farther reasoning proper, it would be manifest, that all the phenomena, both in health and disease, belonging to the various kinds of sputum, consist with the theory above delivered.

